# B-CAN: a resource sharing platform to improve the operation, visualization and integrated analysis of TCGA breast cancer data

**DOI:** 10.18632/oncotarget.21947

**Published:** 2017-10-19

**Authors:** Can-Hong Wen, Shao-Min Ou, Xiao-Bo Guo, Chen-Feng Liu, Yan-Bo Shen, Na You, Wei-Hong Cai, Wen-Jun Shen, Xue-Qin Wang, Hai-Zhu Tan

**Affiliations:** ^1^ Department of Physics and Computer Applications, Shantou University Medical College, Guangzhou, China; ^2^ Joint Institute of Engineering, Sun Yat-Sen University, Carnegie Mellon University, Guangzhou, China; ^3^ Department of Statistical Science, School of Mathematics, Sun Yat-Sen University, Guangzhou, China; ^4^ Southern Research Center for Statistical Science, Sun Yat-Sen University, Guangzhou, China; ^5^ Zhongshan School of Medicine, Sun Yat-Sen University, Guangzhou, China; ^6^ Department of Computer Science, Shantou University, Guangzhou, China; ^7^ Department of Bioinformatics, Shantou University Medical College, Guangzhou, China

**Keywords:** TCGA, breast cancer, data customization, data visualization, private data center

## Abstract

Breast cancer is a high-risk heterogeneous disease with myriad subtypes and complicated biological features. The Cancer Genome Atlas (TCGA) breast cancer database provides researchers with the large-scale genome and clinical data via web portals and FTP services. Researchers are able to gain new insights into their related fields, and evaluate experimental discoveries with TCGA. However, it is difficult for researchers who have little experience with database and bioinformatics to access and operate on because of TCGA’s complex data format and diverse files. For ease of use, we build the breast cancer (B-CAN) platform, which enables data customization, data visualization, and private data center. The B-CAN platform runs on Apache server and interacts with the backstage of MySQL database by PHP. Users can customize data based on their needs by combining tables from original TCGA database and selecting variables from each table. The private data center is applicable for private data and two types of customized data. A key feature of the B-CAN is that it provides single table display and multiple table display. Customized data with one barcode corresponding to many records and processed customized data are allowed in Multiple Tables Display. The B-CAN is an intuitive and high-efficient data-sharing platform.

## INTRODUCTION

Breast cancer is a high-risk heterogeneous disease [1, 2] and complicated biological features, which leads to various complex treatments and clinical outcomes [3]. Understanding the clinical, molecular and genetic basis is crucial for the development of new treatments and preventions for breast cancer [1, 4]. To facilitate researchers in identifying pathogenesis and therapy for breast cancer, the TCGA breast cancer database provides comprehensive 1086 breast cancer patients’ clinical information and 7 types of omics data [5–7]. More specifically, it includes protein, microRNA, gene expression, copy number, and DNA Methylation, along with tissue images [8, 9]. From June 30th, 2016, all the original TCGA data including the breast cancer data can be downloaded from the Genomic Data Commons [9–13] (https://taga-data.nci.nih.gov/docs/publications/tcga/)

Although the TCGA breast cancer database helps researchers accelerate the development of treatments and preventions for breast cancer [14], it could still be challenging for researchers. Massive data lead to time-consuming manual download procedures mainly because a back-end database to handle large-scale datasets may occupy too much memory in the backstage resources. Take the constantly updated breast cancer data as an example, there are about 4099 compressed files with a total size as 6.69 Terabyte (T) by July 6^th^, 2016. Firehose_get, an analysis infrastructure developed by the Broad Institute, requires users to install additional programs before downloading manually, and an automatically update is not available [13, 15]. Furthermore, it is difficult for researchers to organize the TCGA data, especially for those who are unfamiliar with the structure of TCGA database or bioinformatics [16, 17]. For example, various data types could increase the complexity of the TCGA database [5]. It is laborious to unify the file format or structure of the TCGA data provided by different collaborative institutes [5]. As a result, additional processing steps are needed to extract and pre-process data before performing analyses. Some R packages, like TCGA2STAT [13], cgdsr [18], are designed to preprocess the downloaded TCGA data for subsequent analyses [12, 19]. However, few of them give users the flexibility to customize data since a pre-specified set of variables is combined into data matrices. Some important variables (such as follow-up information) are excluded, just as TCGA2STAT does [13]. An error was reported when using the “getTCGA” function in Windows operator system (OS). One possible reason is that TCGA2STATis built on UNIX and Mac OS, not on Windows [20]. Another challenge is the various types of omics data in TCGA database. To address these problems, several integrated multi-dimensional data visualization tools (including the CBioPortal for Cancer Genomics (http://cbioportal.org), RICGAToolbox [21], MEXPRESS [22], etc.) have been proposed to depict the TCGA data in a clear and efficient way. CBioPortal provides a simple yet flexible interface to integrate datasets, intuitive visualization, options, and a web interface. It also provides an R package for statistical computing, CGDSR, to query the cancer Genomic Data Server Web API and return data in a structured format [19]. However, to our knowledge, there are few tools that allow users to handle personal data or non-qualifying customized data together (including CbioPortal).

A user-friendly interface is needed to allow a large community of users with little database skills or knowledge in bioinformatics to customize, visualize and organize the downloaded TCGA breast cancer data and own data. A breast cancer platform (B-CAN) (http://www.bcan.med.stu.edu.cn/) is proposed to provide several practical functions to users, like data customization, data visualization, and private data center, and so on. It is an intuitive and high-efficient sharing platform. By introducing the B-CAN platform, we hope to encourage researchers to make full use of the TCGA breast cancer data, which in turn will bring new insights and breakthroughs in breast cancer research.

## FUNCTIONS AND EXAMPLES

**Figure 1:**
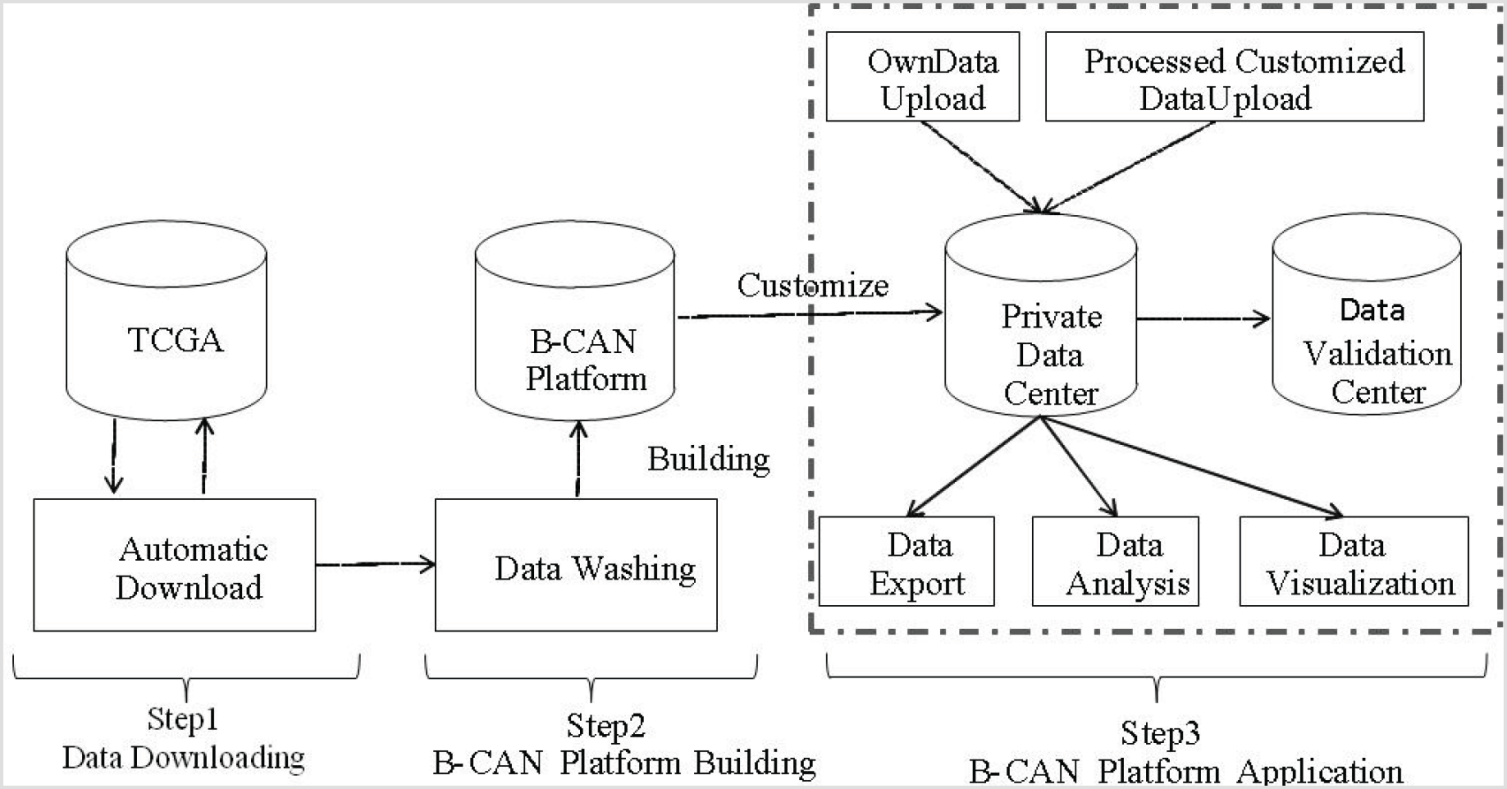
Roadmap of B-CAN platform This figure presents the roadmap of B-CAN platform, which includes data download, platform building, and application. In particular, after automatically downloading data from TCGA breast cancer database, the downloaded data will be cleaned to build the B-CAN platform. Several applications are provided in the B-CAN platform, such as private data center.

All functions of the B-CAN platform are realized through a streamlined 3-part web interfaceadd see Figure 1 here. Specifically, users are guided to perform 1) data customization, 2) Data visualization, 3) Prepared and personal data import. Users have the options to combine one or more downloaded tables from original TCGA platform and select several variables from each table to generate a new database based on their needs. The uploaded personal data do not have the same keyword “barcode”. However, they could be combined together by column after deleting the information “keyword” under the condition that the variables of personal data were same as the customized data. The B-CAN platform automatically generates a series of graphs for a single table or multiple tables. A unique feature of the B-CAN platform is provided for users to upload their private data in the private data center. More detailed instructions on this B-CAN platform operation are provided in the Supplementary Appendix. In addition, a detailed tutorial in the Appendix will show users how to implement series functions in the B-CAN platform.

**Figure 2:**
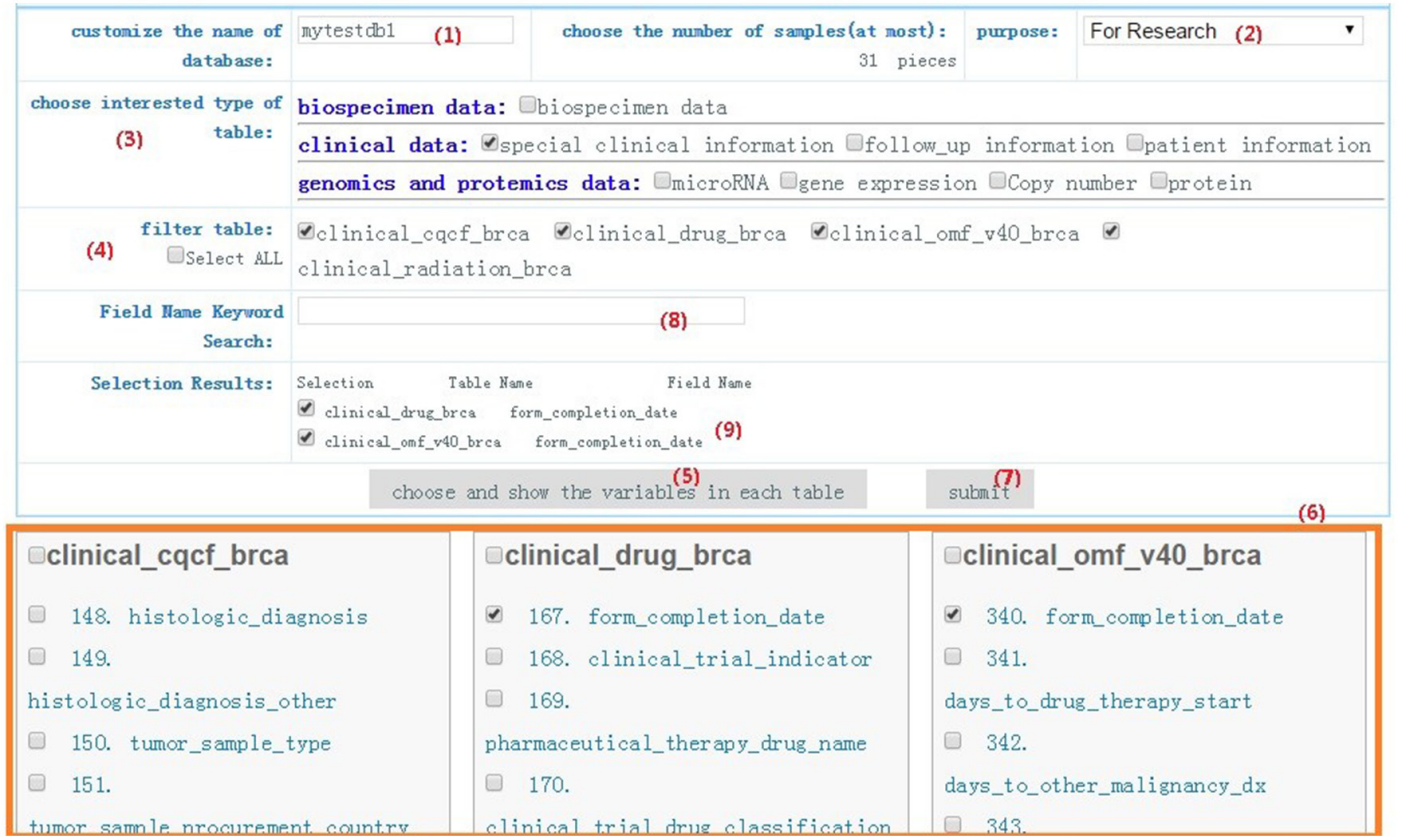
Data customization An example is offered for users to do data customization by following step 1 to 7. After completing the first four steps, variables of interest could be chosen in three optional tables, which contains patient information, follow_up information, and protein information.

For example, some clinicians want to explore whether some proteins and clinical factors of interest influence the survival status of breast cancer patients. They may start by following step 1 to 9 as described in Figure 2 to choose optional three tables including patient information table, follow-up information, and protein table. After giving the name of the new customized database as ‘mytestdb1’ and the purpose of user’s research, user can select one or more interested tables from “choosing interested type of table”. If the interested table has some child tables, users still can select more concrete table from “filter table”. To avoid selecting interested variables from too large number of optional variables, users can also apply some keywords multiple times to narrow down the selections to only those that match. After step 3, 4, 8, users can select the interested variables from the area under the “choose and show the variables in each table” as step 6 showed. And then the searching results are saved in the area of “Selection Results”. As step 9 in Figure 2 showed, interested variables and tables (form_completion_data in clinical_drug_brca or form_completion_data in clinical_omf_v40_brca) are chosen and saved. By clicking “submit”, a new customized database including interested variables from interested tables is formed and saved in the private data center. In this procedure, sample sizes of the customized data with different barcodes will be automatically calculated by the terminal system.

**Figure 3:**
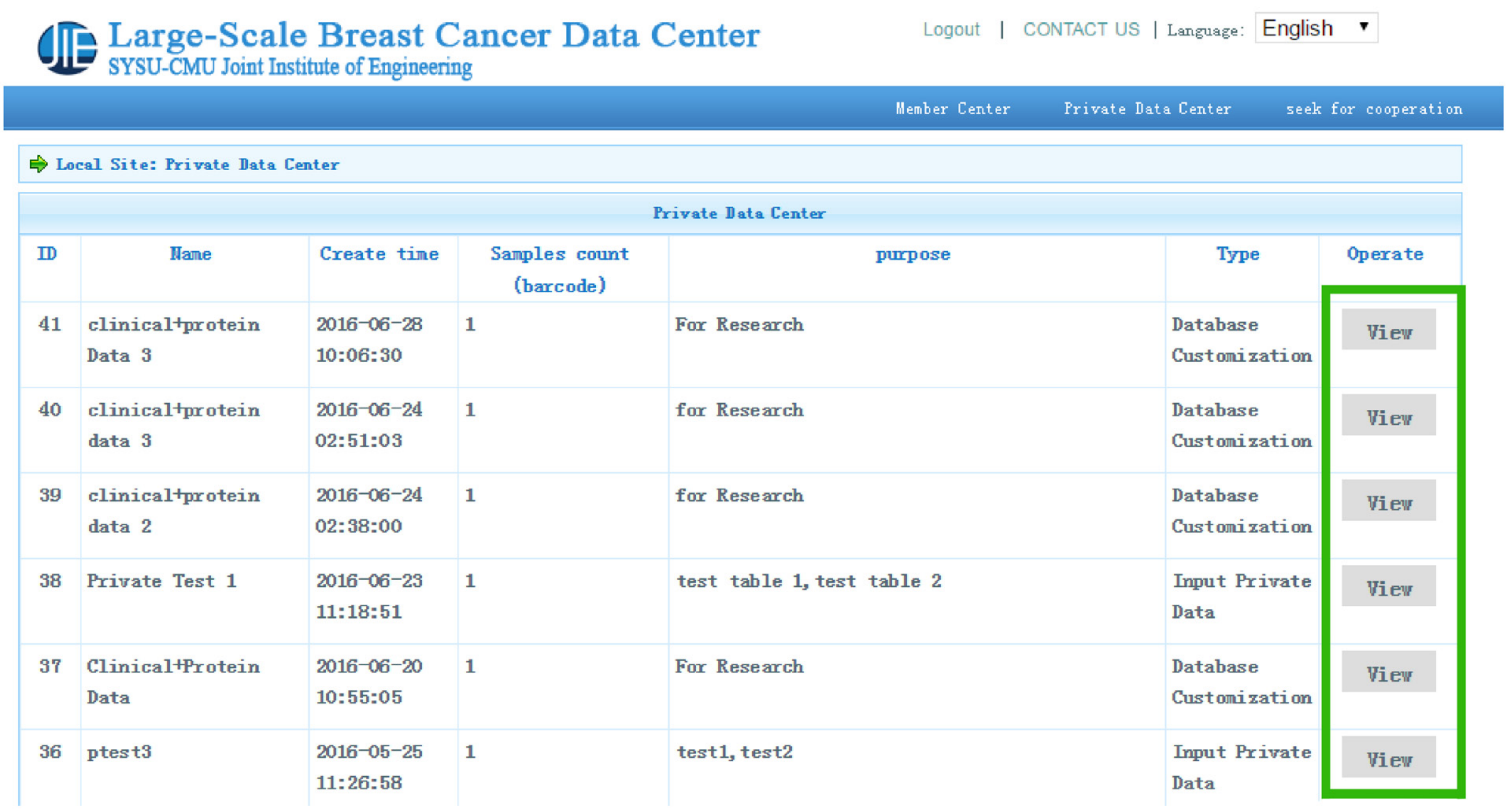
Private data center Two special functions, including single table display and multiple table display, are provided in the private data center.

**Figure 4:**
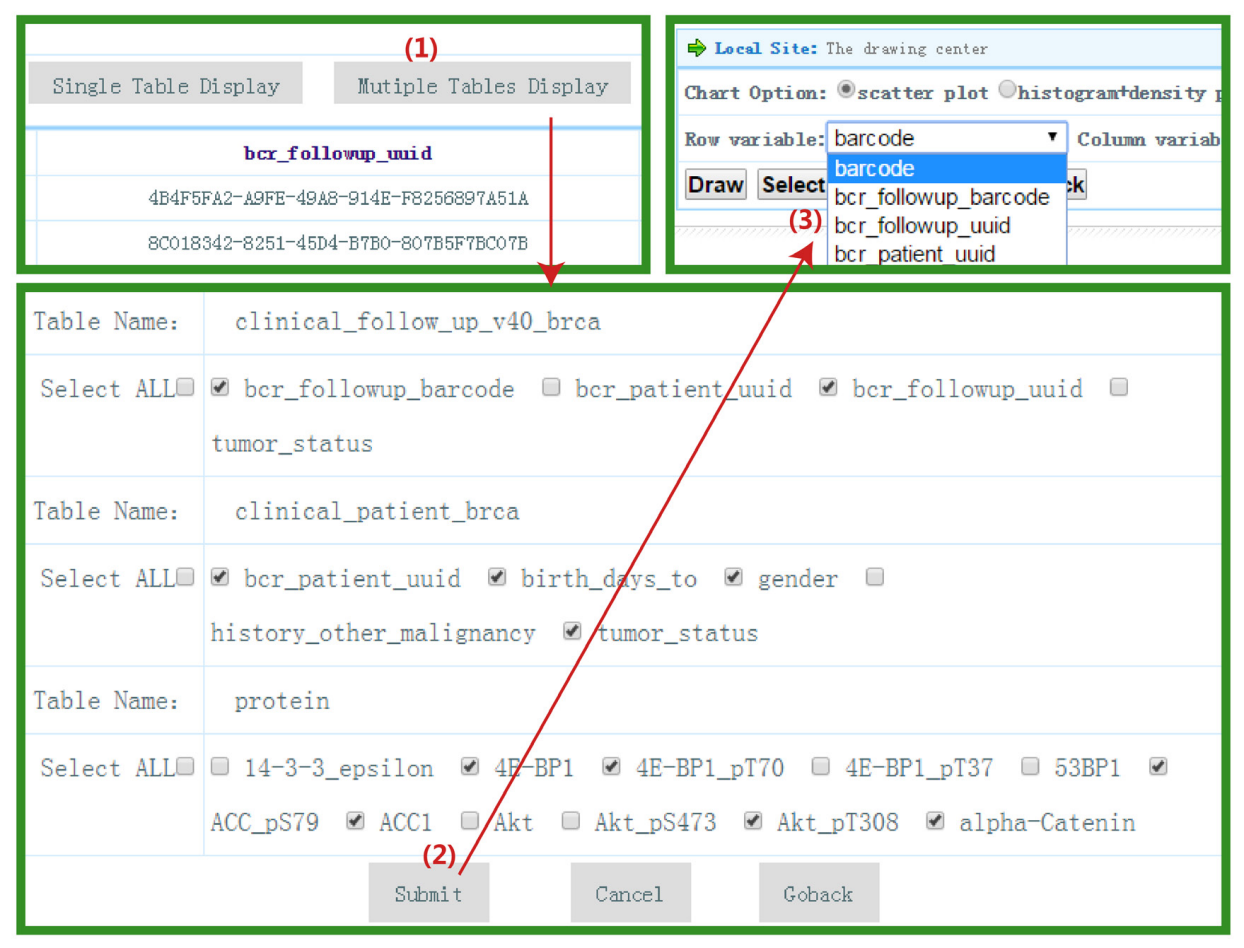
Multiple tables display This figure shows how to do multiple tables display. Users can select variables of interest from different sets of tables and display them.

**Figure 5:**
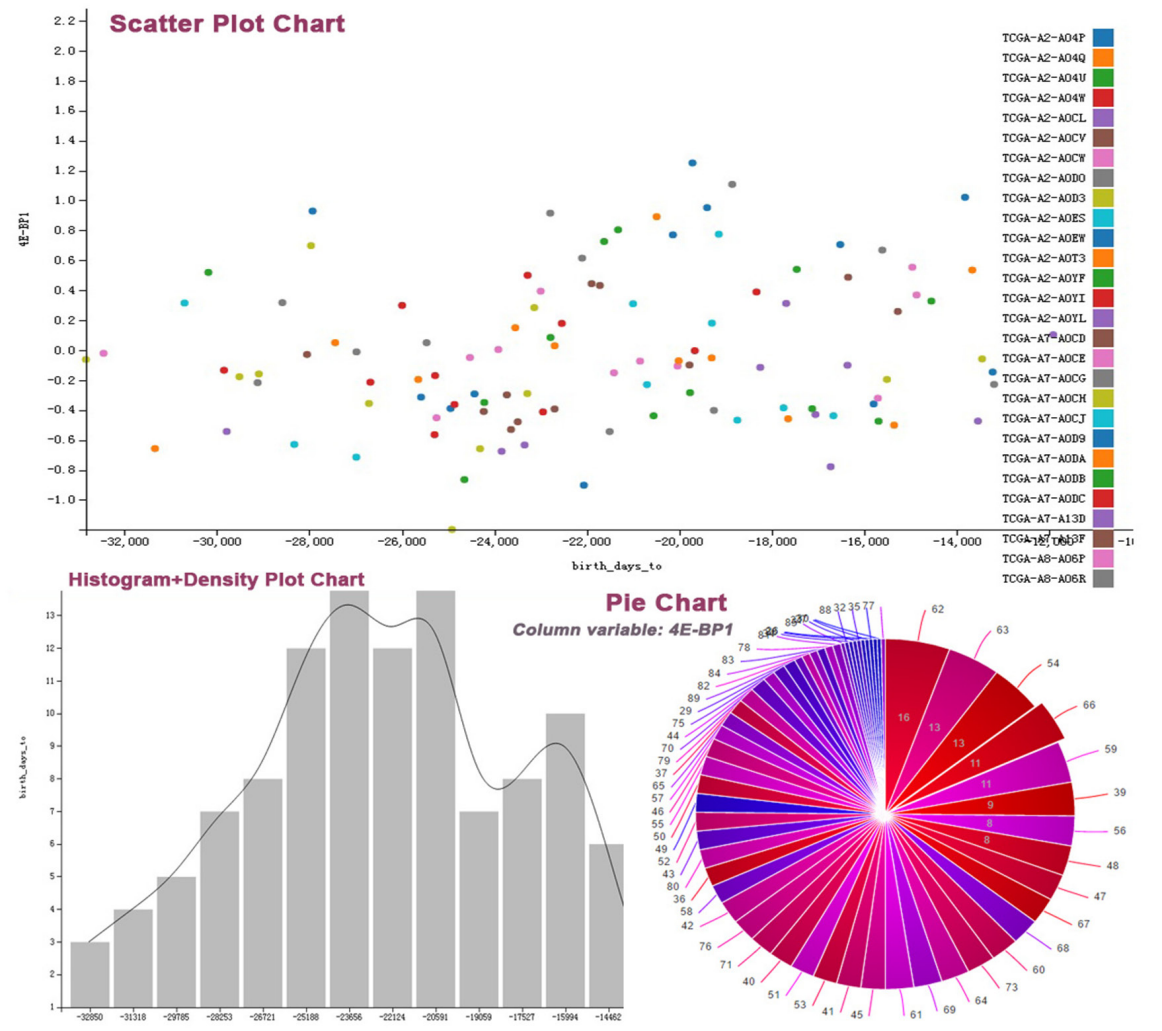
Various figures about data visualization

In the private data center, if users want to depict “4E-BP1” and “14-3-3_epsilon” in a single protein table, they can click the “View” button in the private data center. After choosing a protein table from the drop-down box and clicking the “Single Table Display” button, users can select relevant graphs to describe the relationship between two proteins (see Figure 3). For multiple table display, users can show the relationship between “birth_days_to” in “clinical_patient_brca” table and “4E-BP1” in “protein” table graphically by following steps as shown in Figure 4 after selecting variables of interest from matching tables in “Multiple Tables Display”. Several diagrams (such as scatterplot, histogram plot, and pie chart, etc) are shown in Figure 5.

## DISCUSSION

Breast cancer, as a great health threat, has been widely studied by physicians and researchers from various fields across the world [23]. The TCGA breast cancer database provides a large number of high-dimensional omics data and clinical data for public access. The B-CAN platform, a simple and convenient platform, has features including automatic data update, data customization, private data center, etc. The B-CAN platform may be of particular use for users with little database skills and bioinformatics knowledge. For big original TCGA breast cancer data, the B-CAN is capable of detecting updates automatically. The B-CAN platform offers more flexibility by allowing users to pick variables of interest to generate new databases. Compared to some other TCGA data visualization tools, the B-CAN adopts the D3.js technology with the graphical front-end technique to draw three types of diagrams, including pie chart, scatter plot, and histogram. D3.js provides powerful visualization and interaction functions on huge data sets [24]. In the private data center, users can upload the processed customized data or private data to the private data center. An advanced feature of the B-CAN is Multiple Table Display. It allows users to select variables of interest from multiple related tables and displays the relationship between the selected variables graphically. To those who have a lack of data or want to validate potential factors in pilot experiments, the B-CAN is particularly useful because it provides a richer set of clinical data and 7 types of omics data.

We anticipate several future developments for the B-CAN platform. More types of omics data, including DNA methylation, gene expression data will be covered in the B-CAN platform subsequently. We also plan to add more types of graphs (eg. pathway diagram, heat map, et. al.) to depict data. Further extensions to the cross-omics data or cross-tumor query analysis will be made. Moreover, we will keep revising the B-CAN platform according to users' feedback.

In addition, it is very essential for users to correctly identify independent and significant predictors that are not found in each of their own 7 types of Omics data. We plan to design a convenient way to help users to evaluate the feasibility and validity of their potential significant signals from B-CAN platform and their personal data. For example, if users want to recognize the highly significant predictors from 410 proteins, user can integrate the downloaded summary data of protein from the Integrated Data Analysis and the summary statistics from the univariate own protein data, and then multivariate analysis by some certain statistics method is conducted to detect the potential protein signals.

Consequently, the B-CAN platform provides automatic data downloading, periodic update, data customization, data visualization, and special private data center to users. Users can access and manage the huge B-CAN breast cancer data easily and flexibly without database and bioinformatics knowledge. We encourage scientists from different fields to make use of the rich TCGA dataset, which expectantly will shed light on the future research on breast cancer.

## MATERIALS AND METHODS

The B-CAN runs on Apache server and interacts with the backstage of MySQL database by PHP. The B-CAN platform comprises of comprehensive data types, which includes ‘clinical’, ‘microRNA’, ‘gene expression’, ‘copy number’ and ‘protein’. To illustrate how the B-CAN platform works, we present flow chat of B-CAN in Figure 1. Details of each step are described in four separate subsections as follows.

### Data downloading

Features such as multi-folder, multi-file, and complex depth directory might bring great difficulties in checking updates and downloading sequentially. We here apply TCGA Crawler with the Breadth-First-Search algorithm [25], an improvement of the web crawling tool, to analyze and extract information automatically from the TCGA FTP server (https://tcga-data.nci.nih.gov/tcgafiles/ftp_auth/distro_ftpusers/anonymous/tumor/brca/). The update time of downloaded data files are recorded and thus we can run the updating program regularly based on their last update time. Furthermore, write these pre-selection files into the SQL resource pool to realize the automatic updating function.

### Data customization

Data in the original TCGA file are separated by blanks, ‘\t’ or both them, which might cause the incompatible format problem such as misclassification. Thus, we transform all the downloaded files into the ‘csv’ format. Furthermore, the commas and double quotation marks are cleaned up in the original files to avoid being confused. A table is split into a set of child tables if its fields exceed the maximal number (about 500 fields at most for a chart and about 200 in the type of ‘varchar’ are supported in the MySQL). Tables with the same barcodes in the first row and attributes are combined to enlarge the sample size.

### Data visualization

We utilize the waterfall flow technique to display all the customized data which including the massive tables and fields. For example, a hundred tables are loaded on the screen each time as users scroll down to the end of the file by using this technique. After choosing variables from the customized data, processed customized data or their personal data, users can apply Data Driven Document (D3.js) to graphically display them quickly. The D3.js uses the front-end calculation technique to ensure high working efficiency [25]. When different users operate at the same time, it won’t occupy too much space in the backstage resources.

### Private data center

In the private data center, users can upload their own personal data or processed customized data from the B-CAN platform. For the personal data, users batch upload their own files with file format satisfying the requirements of the platform. A series of security mechanisms in the B-CAN platform provide users’ personal data with a secure environment (firewall and multifactor and multi-layer authentication, etc.). When data from the “Data Customization” step have a one-to-many relationship, users can download the data to the local machine temporarily. After processing the data with right format, users re-upload them to the private data center Consequently, the unified private data center comprised of personal data and two types of customized data (one-to-one relationship customized data and processed customized data). A key feature of the private center is that it provides both Single Table Display and Multiple Tables Display. Personal data and one-to-one relationship customized data are allowed in Single Table Display. Multiple Tables Display can work only for customized data or personal data individually. Although personal data don’t have the same keyword (“barcode”) as customized data, we can still combine personal data and customized data to do multiple table display and graphical display. The main reason is that we can delete the “barcode” under the condition both two data have the same variables. In addition, we can apply personal data to do data mining, and then customized data was treated as external data to validate the model built on the personal data, and vice versa.
